# Cathode Design and Flow Field Optimization in Electrochemical Machining of Square Holes

**DOI:** 10.3390/mi17050578

**Published:** 2026-05-07

**Authors:** Xuesong Liu, Zhen Guo, Fan Du, Guokang Su, Hua Chen, Chuanyun Zhang

**Affiliations:** 1 School of Mechatronic Engineering, Xi’an Technological University, Xi’an 710021, China; liuxuesong1981@sohu.com (X.L.); guozhen_92@163.com (Z.G.); dyf708@126.com (F.D.); suguokang@xatu.edu.cn (G.S.); chenhua126@163.com (H.C.); 2 Inner Mongolia North Heavy Industries Group Corp. Ltd., Baotou 014030, China; 3 Shaanxi Engineering Research Center of Digital Precision Electrochemical Machining, Xi’an 710021, China

**Keywords:** square hole, electrochemical machining (ECM), cathode structure design

## Abstract

To improve the forming quality, precision, and machining stability of square hole structures in high-hardness gun steel (PCrNi_3_MoVA) during electrochemical machining (ECM). A planar cathode bottom design array with liquid holes is innovatively proposed in this paper to achieve uniform distribution of the flow field in discrete bottom machining gaps. The modeling and simulation of the flow field within the ECM gap were carried out using simulation software. A cathode with 25 outlet holes in an array distribution and a profile thickness of 1 mm was designed. However, sparking occurred on the cathode bottom surface during ECM experiments, leading to machining short-circuit. Further analysis and structural optimization were conducted on the sparking area of the cathode bottom surface. The introduction of flow guide grooves on the cathode bottom surface can effectively improve the uniformity of flow field distribution and the stability of the machining process, thereby solving the problem of manufacturing square holes in high-hardness gun steel materials. Finally, under the conditions of an electrolyte pressure of 0.7 MPa, a machining voltage of 12 V, a frequency of 2 kHz, a duty cycle of 60%, and a feed rate of 0.8 mm/min, a square hole with a side length of 10.2 mm was obtained, with a straightness error of ±0.05 mm and a filet radius of 0.38 ± 0.05 mm.

## 1. Introduction

Many square hole structures are widely employed in fields such as artillery and aerospace. Processing methods including stamping, slotting, electric pulse machining, wire electrical discharge machining (WEDM), laser cutting, and ultrasonic-assisted machining can all be used to achieve the fabrication of square holes [1,2,3,4,5]. However, these methods have obvious limitations: slotting requires prefabricated holes; in particular, for high-hardness gun steel, low machining accuracy and severe tool wear are unavoidable. In practical applications, WEDM is incapable of fabricating square blind holes. In addition, electric pulse and laser cutting entail significantly high processing costs and tend to impair the metallic properties of the workpiece [6].

Electrochemical machining (ECM) achieves the target geometry by dissolving the anode material atom by atom [7]. It features relatively simple forming, negligible macroscopic cutting force, and high machining quality [8,9,10]. Researchers have widely adopted finite element numerical analysis in ECM. Rational optimization of the physical fields in the inter-electrode gap can not only guarantee process stability but also improve machining efficiency and surface quality to a certain extent [11,12]. Zhu et al. optimized the flow field in the machining zone by adjusting the inter-electrode gap and electrolyte flow rate [13]. Chen et al. conducted three-dimensional numerical simulations of electrochemical milling accounting for coupled flow and electric fields [14]. They found that the evolution of the anode profile changes the jet reflection pattern, and regulating this pattern can suppress stray corrosion, whereas secondary corrosion cannot be eliminated.

Meanwhile, extensive research has been conducted to improve the stability of square hole machining. Cheng et al. optimized the flow field in the machining zone by adjusting the inter-electrode gap and electrolyte flow rate [15]. Their results indicated that this method improves the dimensional accuracy and processing stability of tapered square holes. Natsu et al. introduced ultrasonic vibration to the tool electrode to improve the speed and accuracy of ECM, investigating the effects of ultrasonic waveform and amplitude on forming quality [16]. Experimental results demonstrated that ultrasonic-assisted ECM achieves the highest material removal rate and favorable machining accuracy. Zhang et al. studied ECM of micro square holes on high-hardness elastic materials using two approaches: single reciprocating machining and a combined method of polyline and loop machining [17]. They found that the combined polyline-loop strategy produced micro square holes with sharp corners, high dimensional accuracy, and excellent machining performance.

Although the above studies have contributed to improving the accuracy and surface quality of micro square holes in ECM through theoretical simulation and auxiliary methods such as vibration, relatively little attention has been paid to the high-precision machining and process stability of macro-scale square holes. Moreover, since the above research requires the assistance of complex methods, it is difficult to ensure the continuous fabrication capability of macroscopic square holes. To further improve the quality accuracy and stability of electrolytic square hole forming, this paper innovatively proposes a discrete small hole structure design on the cathode bottom and an electrolytic machining method with internal injection liquid supply for forming. However, process experiments revealed that the flow field distribution in the square hole direction is non-uniform during the electrolytic machining process, resulting in a short circuit.

## 2. Materials and Principles

### 2.1. Materials

The workpiece material is gun steel (PCrNi_3_MoVA, made in China), which is manufactured via a conventional forging method, and its chemical composition is listed in Table 1.

### 2.2. Principle and Experimental Setup of Square Hole Electrochemical Machining

**Figure 1:**
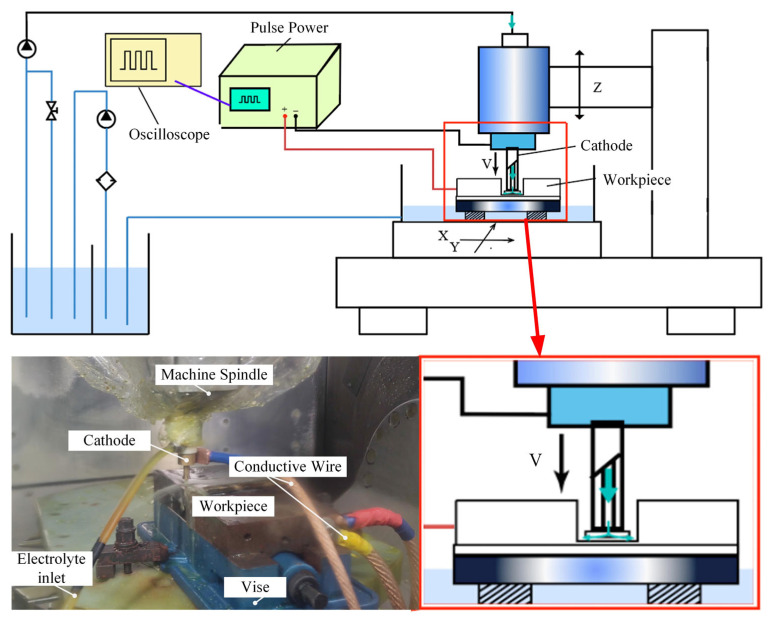
Schematic diagram of the electrochemical machining experimental platform.

Electrochemical machining (ECM) removes workpiece material based on the principle of anodic electrochemical dissolution. Figure 1 shows the schematic diagram of the square hole electrochemical forming principle, illustrating the square hole machining process and electrolyte flow mode. Electrolytes flow out through the inner hole of the cathode and fill the inter-electrode gap, where inter-electrode electrochemical reactions occur. During the reaction, the high-speed flowing electrolyte flushes out electrolytic products and reaction-generated heat from the gap, ensuring the continuous operation of electrochemical machining.

The ECM experimental platform mainly consists of a machine tool, a control system, a fluid supply system, and a machining power supply. The machining process is carried out on a KMC600S five-axis vertical CNC machine tool (KEDE Numerical Control Co., Ltd., Shanghai, China). The control system adopts a GNC62 bus-type numerical control system to achieve rapid machine tool response. The fluid supply system is mainly composed of an electrolytic filtration system and an electrolyte storage tank. The machining power supply provides both pulse and direct current (DC) output modes for ECM, with strong anti-interference capability and high stability.

## 3. Simulation of Physical Field Model in Machining Gap and Cathode Design

### 3.1. Physical Field Model and Parameter Setting

During the entire electrochemical machining process, under the high-speed scouring action of the electrolyte, the relative volumes of gas and solid phases contained in the electrolyte within the machining gap can be neglected. Therefore, the flow field in the inter-electrode gap can be simplified as a single-phase turbulent flow. According to the fluid mechanics principles, for incompressible fluids, without accounting for the effects of gas and solid phases, the flow obeys mass conservation and momentum conservation, and its basic governing equations are as follows:

Mass Conservation Equation:


(1) $$\frac{\partial \rho }{\partial t}+\frac{\partial \left(\rho u\right)}{\partial x}+\frac{\partial \left(\rho v\right)}{\partial y}+\frac{\partial \left(\rho w\right)}{\partial z}=0$$

Momentum Conservation Equation:

(2) $$\frac{\partial \left(\rho u_{i}\right)}{\partial t}+\frac{\partial \left(\rho u_{i}u_{j}\right)}{\partial x_{j}}=-\frac{\partial p}{\partial x_{i}}+\frac{\partial \tau_{ij}}{\partial x_{j}}+\rho g_{i}+F_{i}$$

where ρ is the fluid density, $x_{i}$, $x_{j}\;$ are coordinate tensors, $u_{i},u_{j}$ are velocity tensors, *p* is the pressure, $\tau_{ij}$ is the viscous stress tensor, $\rho g_{i}$ is the gravitational body force, and $F_{i}$ denotes other body forces.

The most widely used mathematical model for describing turbulent flow is the standard *k* − ε two-equation model, which consists of a transport equation for turbulent kinetic energy (*k*) and a transport equation for turbulent dissipation rate (ε). The k equation characterizes the transport and dissipation of turbulent energy, while the ε equation describes the evolution of the turbulent energy dissipation rate. These two equations allow the calculation of parameters such as flow velocity and turbulence intensity in turbulent motion, facilitating a clearer understanding of the characteristics and mechanism of turbulent flow.

In this study, the standard *k* − ε two-equation model is adopted for the numerical simulation of electrolyte flow.

(3) $$\frac{\partial \left(\rho \varepsilon \right)}{\partial t}+\frac{\partial \left(\rho kv_{i}\right)}{\partial x_{i}}=\frac{\partial }{\partial x_{j}}\left[\left(\mu +\frac{\mu t}{\rho k}\right)\frac{\partial k}{\partial x_{i}}\right]+G_{k}-\rho \varepsilon \frac{\partial \left(\rho \varepsilon \right)}{\partial t}+\frac{\partial \left(\rho \varepsilon v_{i}\right)}{\partial x_{i}}=\;\frac{\partial }{\partial x_{j}}\left[\left(\mu +\frac{\mu t}{\sigma k}\right)\frac{\partial \varepsilon }{\partial x_{j}}\right]+\frac{C_{1\varepsilon }\varepsilon }{k}G_{k}-C_{2\varepsilon }\rho \frac{\varepsilon^{2}}{k}$$

(4) $$G_{k}=\mu_{t}\left(\frac{\partial v_{i}}{\partial x_{j}}+\frac{\partial vj}{\partial x_{i}}\right)$$

where $G_{k}$ is the turbulent kinetic energy generated by mean velocity gradients, $\mu_{t}$ is the turbulent viscosity, and ε is the turbulent kinetic energy dissipation rate.

In COMSOL 5.1 software, these parameters adopt default constant values: $C_{1\varepsilon }$ = 1.44, $C_{2\varepsilon }$ = 1.92. The turbulent Prandtl numbers for turbulent kinetic energy k and dissipation rate *ε* are $\sigma_{k}$ = 1, $\sigma_{\varepsilon }$ = 1.3, respectively.

Since the parameters during machining remain constant over time, other fixed parameters are listed in Table 2.

### 3.2. Flow Field Design at the Cathode Outlet

**Figure 2:**
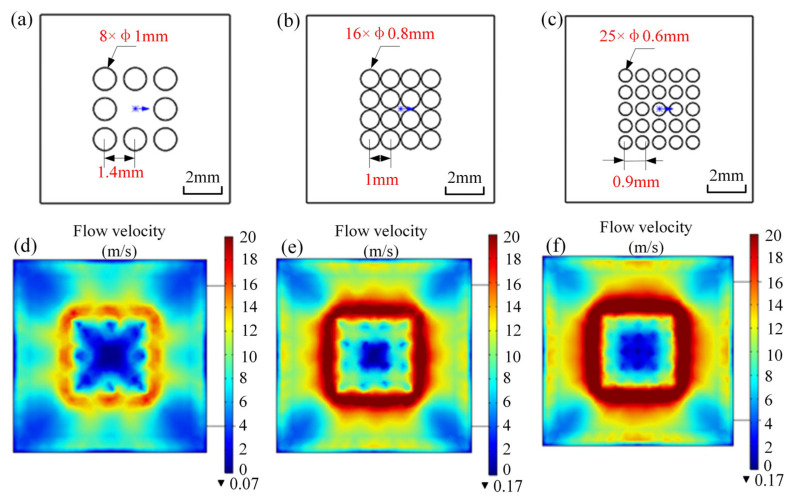
Velocity distribution in the machining gap with different numbers of outlet holes. (**a**) 8-Holes; (**b**) 16-Holes; (**c**) 25-Holes; (**d**) Flow field distribution of 8-Holes; (**e**) Flow field distribution of 16-Holes; (**f**) Flow field distribution of 25-Holes.

To alleviate the non-uniform flow velocity distribution along the square hole edges on the workpiece surface inside the machining gap, the arrangement of outlet holes on the cathode end face was redesigned. A square array of circular orifices was adopted to make the flow path of the electrolyte toward each side of the square hole as uniform as possible. Considering the practical machining constraints and to avoid insufficient material dissolution and residual layers induced by oversized outlet holes, three hole diameters of 1 mm, 0.8 mm, and 0.6 mm were designed. Correspondingly, the cathode machining end face was configured with 8, 16 and 25 outlet holes, as illustrated in Figure 2a–c.

The number of outlet holes on the cathode directly determines the flow velocity and distribution of electrolytes within the machining gap. An insufficient number of outlet holes causes inadequate electrolyte supply in the machining zone, thereby deteriorating the flow field stability. Conversely, an excessively large number of holes leads to unnecessary machining costs. Therefore, to achieve a reasonable cathode structure, it is essential to select a proper number of outlet holes according to finite element simulation results. For the aforementioned structural schemes, the simulated distributions of electrolyte flow velocity and pressure are presented in Figure 2d–f. It is observed that with an increase in the number of outlet holes, the gradient of pressure distribution and the electrolyte flow velocity on the workpiece surface are gradually optimized, accompanied by a reduction in low-velocity regions. The eight-hole scheme in Figure 2d shows generally low flow velocity and pressure over the entire domain. Local regions with extremely low velocity and pressure fail to evacuate machining byproducts in a timely manner, which may induce short-circuiting during ECM. In comparison, the 16-hole and 25-hole schemes deliver more favorable velocity and pressure distributions.

**Figure 3:**
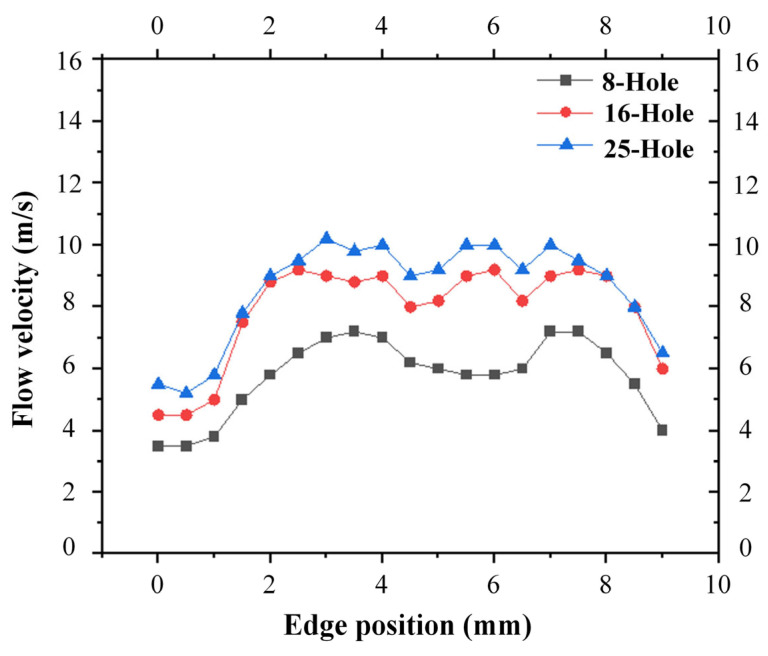
Line graph of flow velocity distribution along the square hole edge.

Simulation results indicate that the flow velocity distributions along the four edges of the square hole are nearly identical. Accordingly, edge a can be taken as a representative to characterize the velocity distribution of all four edges. The edge flow velocity data corresponding to the 8-hole, 16-hole and 25-hole cathode layouts are plotted in the curve diagram, as shown in Figure 3.

For the eight-hole layout, the flow velocity at both the midpoint and corners of the square hole edge is relatively low, with a minimum corner velocity of 3.19 m/s. Such a low velocity cannot guarantee stable electrochemical machining (ECM). For the 16-hole layout, the flow velocity at the edge midpoint increases and exhibits better uniformity compared with the 8-hole scheme. Nevertheless, the minimum velocity at the corners is only 4.24 m/s, which still cannot satisfy the requirement for adequate removal of electrolytic machining byproducts.

In the 25-hole layout, the flow velocity at the edge midpoint achieves a high degree of uniformity, and the velocity along the entire edge exceeds 5 m/s. This effectively mitigates the adverse effect of workpiece surface passivation and improves the overall machining quality. To satisfy practical machining demands, the 25-hole cathode configuration is selected as the final tool electrode scheme.

Although the corner flow velocity of the 25-hole structure is above 5 m/s, it remains lower than that in other regions of the machining gap. Considering that the electric field concentration at the sharp corners of the square hole may induce tip discharge, the cathode structure will be further optimized on the basis of preliminary machining experiments and numerical simulations.

## 4. Experimental Results and Discussion

### 4.1. Experimental Verification and Analysis

**Figure 4:**
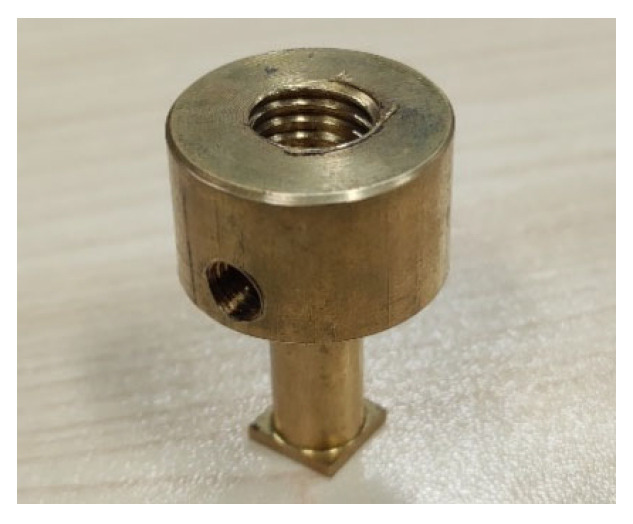
Cathode structure.

Based on the physical field model analysis, the corresponding cathodes were designed and manufactured, as shown in Figure 4. Experimental investigations on square-hole ECM were performed on the established test platform. The workpiece material was high-hardness gun steel, and the preset machining depth was 10 mm. Comparative experiments were conducted to analyze the effects of four structural schemes on the machining accuracy of electrochemically machined square holes. The adopted experimental parameters are summarized in Table 3.

**Figure 5:**
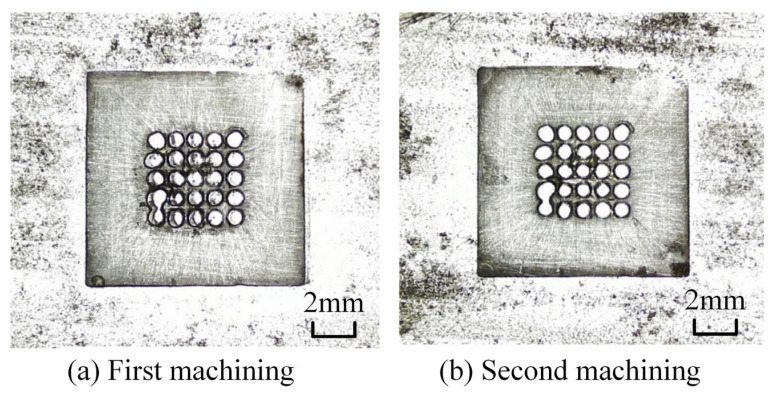
Square hole machined with the original cathode.

Machining experiments were performed in accordance with the predetermined experimental parameters. In repeated trials, spark discharge and electrical breakdown occurred between the cathode tool and workpiece anode at approximately 8 s after machining initiation, resulting in a short circuit and forced termination of the experiment. The machined surface morphology is presented in Figure 5. It can be clearly observed that sparking occurs at the middle corner positions of the square hole. This phenomenon can be attributed to the following reason: during ECM, electrolytic byproducts gradually accumulate within the inter-electrode gap, while the electrolyte flow fails to flush away these byproducts in a timely manner at the sharp corners of the square hole.

**Figure 6:**
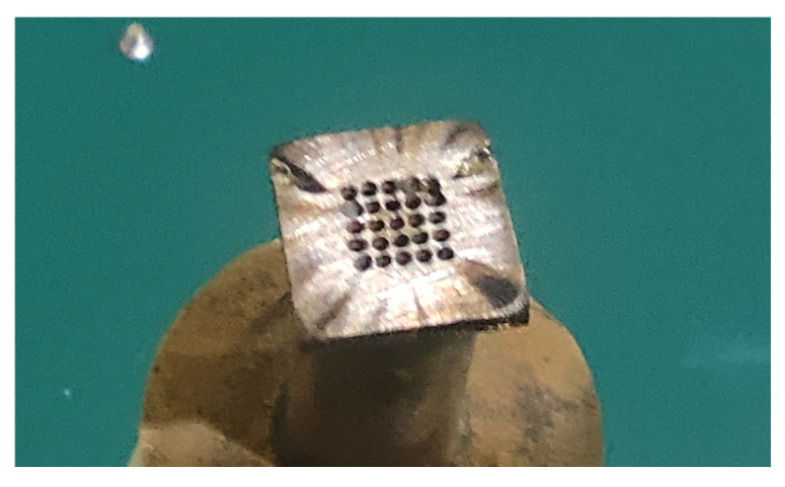
Spark Marks on the Cathode Profile.

It is evident that the original cathode design suffers inherent drawbacks in practical machining. Combined with the spark traces observed on the cathode surface (Figure 6), it can be confirmed that electrolytic byproducts are difficult to discharge from the outlet holes on the cathode bottom toward the middle corners of the square hole. To realize smooth discharge of electrolytic byproducts in the middle region of the cathode profile, diversion grooves are introduced to facilitate the removal of machining products.

### 4.2. Optimization of Cathode Surface Structure

**Figure 7:**
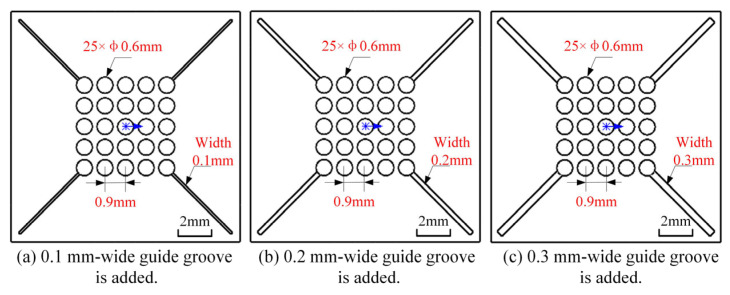
Cathode design with added guide grooves.

Considering that the short-circuit phenomenon of the original cathode is caused by the failure of electrolytic byproducts to be discharged in a timely manner, which stems from the inadequate outflow of electrolyte within the machining gap, diversion grooves with widths of 0.1 mm, 0.2 mm, and 0.3 mm were designed to perform flow field simulation analysis in the machining gap. The modified structures of the three cathode profile designs are presented in Figure 7.

**Figure 8:**
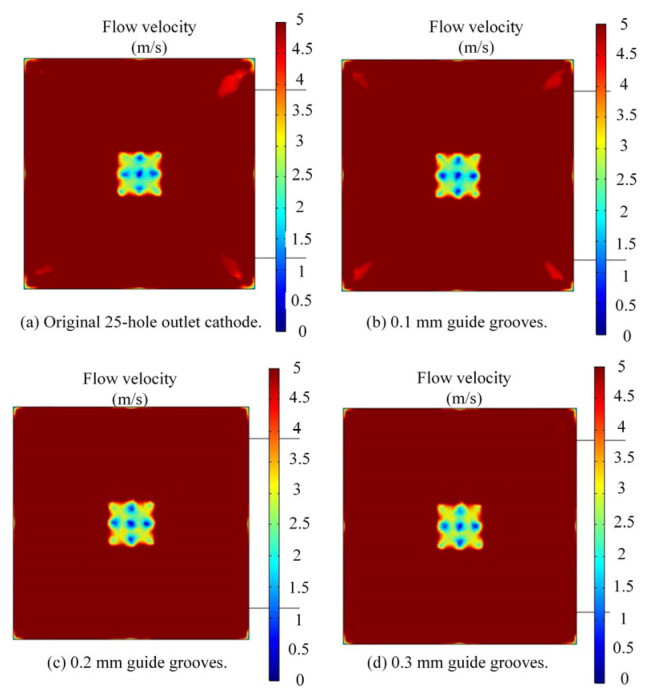
Simulated contour plots of electrolyte velocity on the cathode and workpiece surfaces.

**Figure 9:**
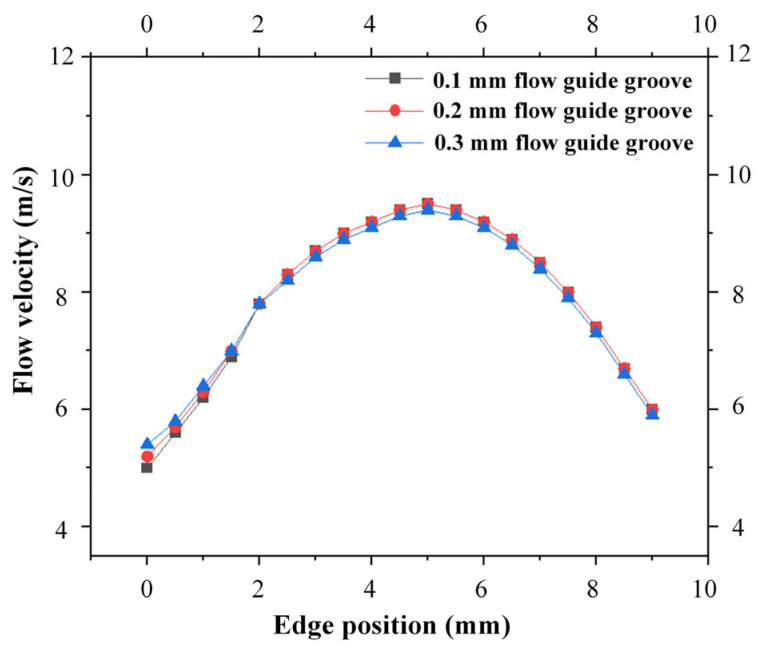
Simulated flow velocity along the square hole edge.

The three modified cathode structures still adopted the flow field simulation parameters listed in Table 2. Meanwhile, to better evaluate the flow velocity, the velocity contour range on the workpiece anode surface was set to 0–5 m/s, and the simulation results are presented in Figure 8 below. It can be observed that for the original cathode, there is a region near the middle corners of the outlet holes on the workpiece anode surface where the electrolyte velocity is lower than 5 m/s, which is consistent with the sparking positions observed in the square hole ECM experiments. After adding diversion grooves, the area with electrolyte velocity below 5 m/s is reduced. Furthermore, as the width of the diversion grooves increases, this low-velocity area tends to shrink further. When the width of the diversion grooves reaches 0.2 mm, the electrolyte velocity in all regions inside the square hole profile near the outlet holes on the anode surface exceeds 5 m/s. Figure 9 shows the simulated line graphs of edge flow velocity under different diversion groove widths. It is evident that increasing the width of the flow guide grooves has a minimal impact on the edge flow velocity. Therefore, adopting flow guide grooves with a width of 0.2 mm is sufficient to ensure the timely discharge of electrolytic byproducts in the ECM gap.

### 4.3. Modified Experiment Results and Discussion

**Figure 10:**
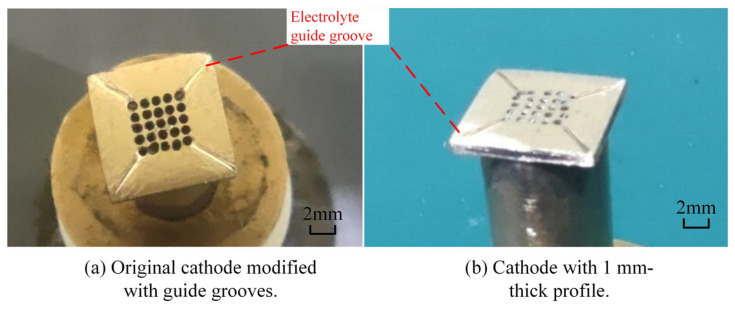
Structures of modified cathodes.

The original cathode modified by adding 0.2 mm-wide flow guide grooves is illustrated in Figure 10a. Three types of cathode modifications were employed for machining experiments: reducing the thickness of the cathode profile from 1.5 mm to 1 mm, chamfering a 0.5 mm filet at the sharp corners of the cathode bottom, and chamfering 0.5 mm edges at the cathode bottom, as presented in Figure 10b.

The experimental parameters for the electrochemical machining of square holes were determined and are listed in Table 4. To fabricate square holes with high precision and efficiency, experimental verification was conducted via electrochemical machining of square holes with a machining depth of 10 mm. Each set of parameters was tested three times.

#### 4.3.1. Influence of Pulse Frequency on Dimensions of Square Holes

**Figure 11:**
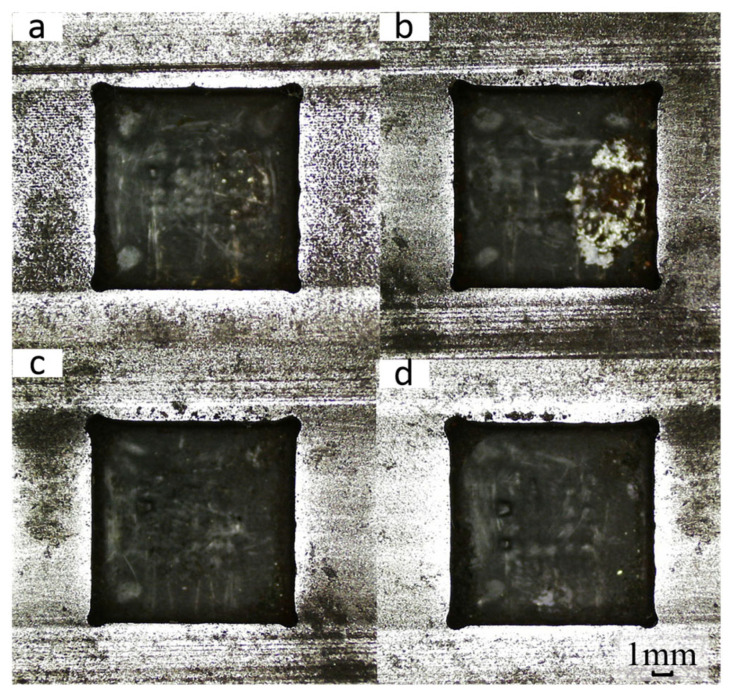
Machining square holes at different power supply frequencies. (**a**) 0.5 kHz; (**b**) 1 kHz; (**c**) 1.5 kHz; (**d**) 2 kHz.

To study the influence of power supply frequency on the forming accuracy of square holes, the power supply frequency was controlled as a single variable while maintaining other test parameters constant (including a voltage of 21 V, electrolyte hydraulic pressure of 0.5 MPa, duty cycle of 70%, and feed rate of 0.8 mm/min). This experimental design aimed to explore the specific impact of different frequencies on the machining and forming process. Different power supply frequencies (0.5 kHz, 1 kHz, 1.5 kHz, and 2 kHz) were set to conduct electrolytic machining of square holes, and the processed square holes are shown in Figure 11.

It is obvious that a metamorphic layer caused by instantaneous short circuits exists on the bottom surface of the square holes when the power supply frequency is 0.5 kHz and 1 kHz. Particularly at a power frequency of 1 kHz, a distinct white area (indicating severe metamorphism) is observed on the workpiece surface. In contrast, the white area on the workpiece surface gradually decreases when the power supply frequency is increased to 1.5 kHz and 2 kHz. This phenomenon indicates that selecting an appropriately higher power supply frequency is beneficial for electrolytic machining of square holes.

**Figure 12:**
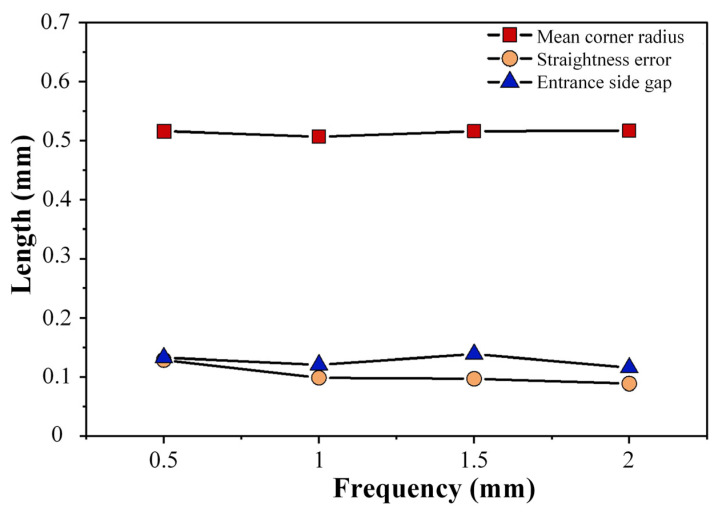
Measurement data of machining results at different frequencies.

In order to determine the optimal parameters of power supply frequency more specifically, the line diagram (Figure 12) drawn according to the measurement data is shown below. According to the line diagram, it can be seen that with the increase in power supply frequency, the corner radius at the tip corner of the square hole changes little, and it can be seen that the power supply frequency has little effect on the corner radius. The straightness error of the edge of the square hole decreases with the increase in power supply frequency, showing a negative correlation trend as a whole. The minimum process parameter of the inlet side clearance is 0.11 mm at 2 kHz. According to the theoretical analysis, the increase in the power supply frequency and the corresponding shortening of the pulse period during electrolytic machining will increase the number of discharges per unit time, resulting in an increase in the removal rate of anode material. Therefore, the power supply frequency should be 2 kHz with better processing stability and higher forming accuracy.

#### 4.3.2. Influence of Pulse Duty Cycle on Dimensions of Square Holes

**Figure 13:**
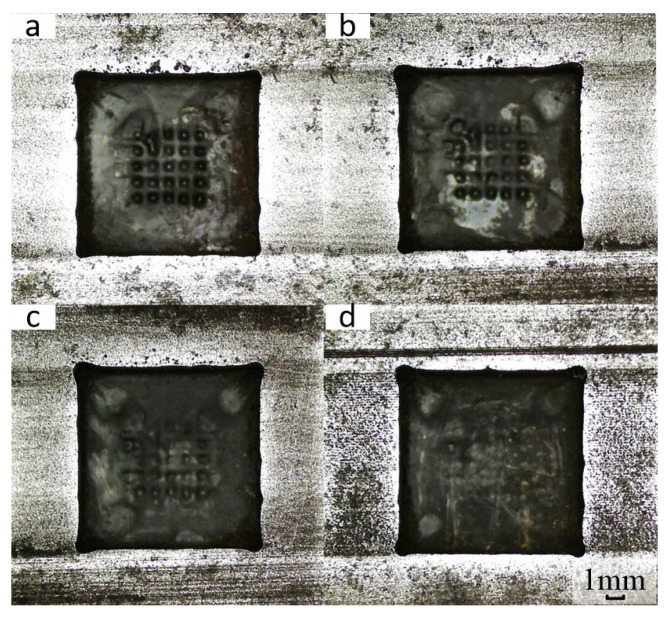
Machining square holes with different duty cycles. (**a**) 40%; (**b**) 50%; (**c**) 60%; (**d**) 70%.

To investigate the influence of duty cycle on the forming accuracy of square holes, the duty cycle was controlled as a single variable while keeping other processing parameters constant, including a processing voltage of 21 V, electrolyte hydraulic pressure of 0.5 MPa, power supply frequency of 2 kHz, and feed rate of 0.8 mm/min. Square holes machined with different duty cycles (40%, 50%, 60%, and 70%) are presented in Figure 13. As shown by the experimental results, stray corrosion during machining is relatively slight under low duty cycle parameters. Specifically, when the duty cycle is 40% and 50%, the contour effect of the square hole edges is superior; however, an instantaneous short-circuit white area appears on the bottom surface of the square holes due to excessively low current density.

**Figure 14:**
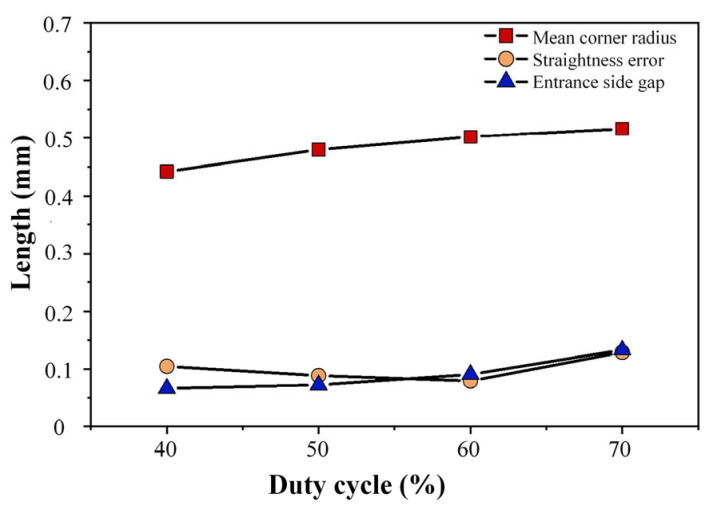
Measurement data of machining results at different duty cycles.

According to the line chart (Figure 14), as the duty cycle increases, the corner radius at the tip of the square hole also increases, showing an overall positive correlation trend. An increase in the duty cycle extends the discharge duration, which in turn increases the material removal rate and intensifies stray corrosion. Meanwhile, the straightness error of the square hole edge decreases with the increase in the duty cycle but rises to the maximum when the duty cycle reaches 70%. Theoretically, a higher duty cycle can prolong the electrolyte renewal time and thereby improve the square hole machining quality. Based on the aforementioned analysis, a duty cycle of 60% is selected as the optimal parameter for square hole electrochemical machining.

#### 4.3.3. Influence of Pulse Voltage on Dimensions of Square Holes

**Figure 15:**
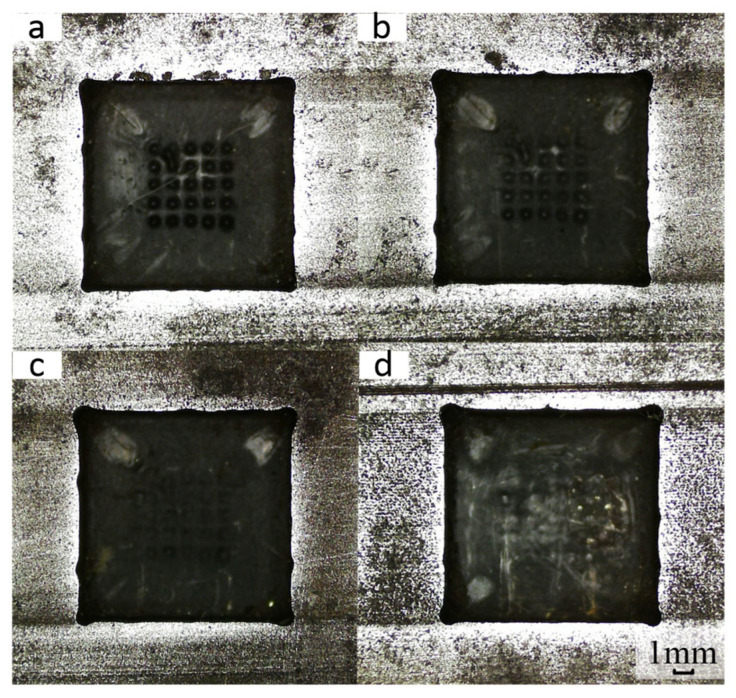
Machining square holes at different processing voltages. (**a**) 12 V; (**b**) 15 V; (**c**) 18 V; (**d**) 20 V.

To explore the effect of machining voltage on the forming quality of square holes, other parameters were kept constant: electrolyte pressure of 0.5 MPa, power supply frequency of 2 kHz, duty cycle of 70%, and feed rate of 0.8 mm/min. Square holes machined at voltages of 12 V, 15 V, 18 V and 21 V are presented in Figure 15. The results show that all four voltage conditions yield good surface quality on the bottom of the square holes, with no obvious metamorphic layer observed. As the machining voltage increases, the corner radius at the square hole tips increases significantly, accompanied by an overall expansion of the contour profile. This is attributed to the fact that a higher machining voltage raises the current density between the cathode and anode, strengthens the material removal rate of gun steel, and consequently aggravates electrochemical corrosion of the workpiece.

**Figure 16:**
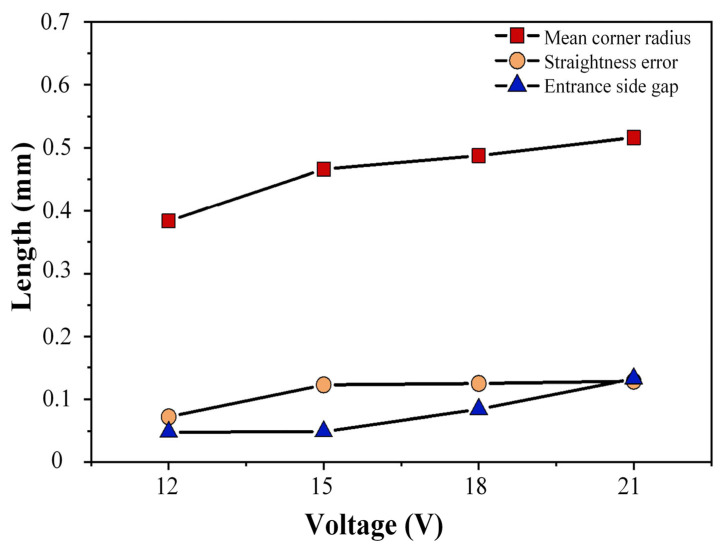
Measurement data of machining results at different processing voltages.

Figure 16 presents the line chart plotted from the measured data of square holes machined at different voltages. As shown in the chart, the corner radius at the sharp corners of the square hole increases obviously with rising machining voltage, demonstrating an overall positive correlation. The edge straightness error reaches its minimum at 12 V and changes slightly when the voltage exceeds 15 V. The inlet side clearance also follows a positive correlation trend; a notable rise in machining voltage enlarges the machining gap.

At a machining voltage of 12 V, the corner radius, edge straightness error, and inlet side clearance all achieve the optimal dimensional accuracy, which can satisfy the stable electrochemical machining of square holes. Accordingly, 12 V is selected as the optimal machining voltage parameter for square hole fabrication.

**Figure 17:**
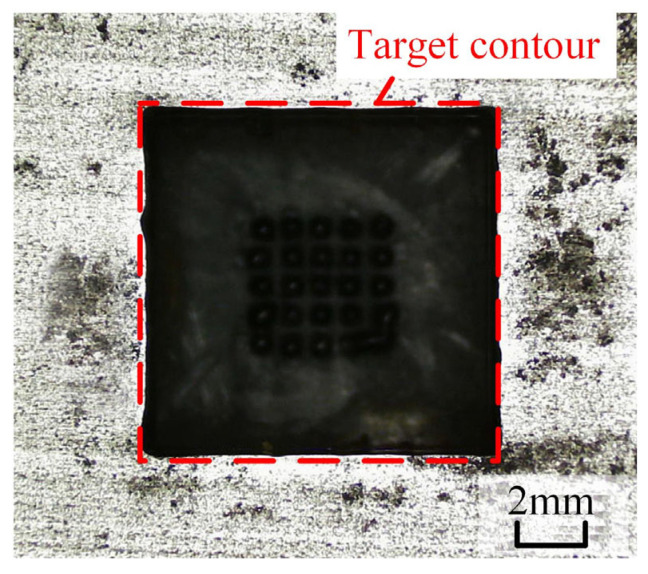
Square hole machined by electrochemical machining.

The optimal process parameters for the electrochemical machining (ECM) of square holes were determined and are summarized in Table 5. To fabricate square holes with high precision and efficiency, experimental verification was conducted by performing electrochemical machining of square holes with a preset machining depth of 10 mm. The final machined square hole is presented in Figure 17.

As can be observed from Figure 11, the machined square hole exhibits excellent profile straightness, small corner radii at the sharp positions, and stable electrochemical machining (ECM) behavior, with high forming accuracy. Measurements were performed on the electrochemically machined square hole structure, and the results are summarized in Table 6. The corner radius of the square hole is 0.38 ± 0.05 mm, the straightness error is 10.2 ± 0.05 mm, and the inlet side clearance is 0.1 mm. All measured dimensions meet the preset design requirements.

## 5. Conclusions

Aiming at the forming quality, accuracy, and stability of square holes in electrochemical machining (ECM), a physical model of the flow field in the machining gap was established. The cathode structure was optimized through numerical calculation and verified by experimental investigation. The main conclusions are as follows:

1. Increasing the number of inlets on the bottom end face of the cathode can effectively enhance the flow velocity of the electrolyte, thereby improving the distribution of the flow field in the machining gap and optimizing the flow field uniformity.
2. Compared with other cathode structures, the design with a 0.5 mm chamfer at the bottom edge of the cathode results in a smaller corner radius of the square hole, a smaller inlet side gap, and better uniformity of the edge profile. Under the conditions of an electrolyte pressure of 0.7 MPa, a machining voltage of 12 V, a frequency of 2 kHz, and a duty cycle of 60%, the machined square hole has a side length of 10.2 mm, a straightness error of ±0.05 mm, and a corner radius of 0.38 ± 0.05 mm, all of which meet the high-precision machining requirements.
3. Adding guide grooves at the four corners of the cathode can effectively improve the flow velocity of the electrolyte in the machining gap, avoid the accumulation of electrolytic byproducts, and thus reduce the occurrence of short circuits and improve the stability of the machining process.

## Figures and Tables

**Table 1:** Main chemical composition of PCrNi_3_MoVA (Wt.%).

Composition	C	Si	Mn	P	Cr	Ni	Cu	Mo	V
Min	0.34	0.17	0.25	0	1.2	3	0	0.35	0.1
Max	0.41	0.37	0.5	0.02	1.5	3.25	0.2	0.45	0.25

**Table 2:** Parameter Settings.

Simulation Parameters	Set Value
Inlet pressure/MPa	0.8
Outlet pressure/MPa	0.101325
Temperature/°C	25
Machining gap/mm	0.2
Dynamic viscosity/Pa·s	0.000899
Electrolyte density/kg·m^−3^	1100
Equilibrium potential/V	−0.441

**Table 3:** Preliminary experiment parameters.

Parameters	Value
Electrolyte	1.5 mol/L NaNO_3_
Temperature	25 °C
Machining gap	0.2 mm
Electrolyte pressure	0.7 Mpa
Machining voltage	15 V
Frequency	2 kHz
Duty cycle	60%
Feed rate	0.8 mm/min

**Table 4:** Experimental parameters.

Parameters	Value
Electrolyte pressure/MPa	0.7
Machining voltage/V	12, 15, 18, 21
Frequency/kHz	0.5, 1, 1.5, 2
Duty cycle	40%, 50%, 60%, 70%
Feed rate/mm·min^−1^	0.8

**Table 5:** Optimal process parameters.

Parameters	Value
Electrolyte pressure/MPa	0.7
Machining voltage/V	12
Frequency/kHz	2
Duty cycle	60%
Feed rate/mm·min^−1^	0.8

**Table 6:** Measurement data of the square hole.

Measurement Item	Value/mm
Mean corner radius	0.38 ± 0.05
Straightness error	10.2 ± 0.05
Entrance side gap	0.1

## Data Availability

Data are contained within the article.

## References

[1] Hu J., Zhang Y. (2022). NGAT: Attention in breadth and depth exploration for semi-supervised graph representation learning. Front. Inf. Technol. Electron. Eng..

[2] Machno M. (2020). Investigation of the Machinability of the Inconel 718 Superalloy during the Electrical Discharge Drilling Process. Materials.

[3] He B., Ying L., Li X., Hu P. (2016). Optimal design of longitudinal conformal cooling channels in hot stamping tools. Appl. Therm. Eng..

[4] Lian H., Zhang L., Chen X., Deng C., Mo Y. (2023). Design of a Template-Based Electrophoretically Assisted Micro-Ultrasonic Machining Micro-Channel Machine Tool and Its Machining Experiment. Micromachines.

[5] Koshy P., Tovey J. (2011). Performance of electrical discharge textured cutting tools. CIRP Ann..

[6] Huang J., Liu X., Zhang H., Zhu T., Shi H. (2026). Challenges and optimization progress in high aspect ratio micro-hole formation for high-frequency and high-speed PCBs: A review. Precis. Eng..

[7] Wang D., Li J., Bin H., Zhu D. (2019). Analysis and control of inter-electrode gap during leveling process in counter-rotating electrochemical machining. Chin. J. Aeronaut..

[8] Zhang C., Xu Z., Zhang J. (2020). Surface integrity of holes machined by electrochemical discharge drilling method. CIRP J. Manuf. Sci. Technol..

[9] Lu J., Zhan S., Liu B., Zhao Y. (2022). Plasma-enabled electrochemical jet micromachining of chemically inert and passivating material. Int. J. Extrem. Manuf..

[10] An L., Wang D., Zhu D. (2022). Combined electrochemical and mechanical polishing of interior channels in parts made by additive manufacturing. Addit. Manuf..

[11] Liu W., Kunieda M., Luo Z. (2021). Three-dimensional simulation and experimental investigation of electrolyte jet machining with the inclined nozzle. J. Mater. Process. Technol..

[12] Zhang C., Zhang Y., Chen X., Yao J., Li J., Su G., Zhao R. (2020). Investigation of flow fields during the electrochemical machining of variable-section pit arrays. J. Mater. Process. Technol..

[13] Zhu D., Zhu D., Xu Z., Xu Q., Liu J. (2010). Investigation on the flow field of W-shape electrolyte flow mode in electrochemical machining. J. Appl. Electrochem..

[14] Chen X., Deng Z., Zhang J., Ye Z., Zhang Y. (2025). Regulating electric field with electrode rotation to enhance surface quality in sinking electrochemical milling of groove. J. Mater. Process. Technol..

[15] Cheng L., Chen X., Ye Z., Zhang Y. (2024). Advancing electrochemical drilling process via coupling of flow field and electric field in pulsating state generated by a novel tube tool. Chin. J. Aeronaut..

[16] Natsu W., Nakayama H., Yu Z. (2012). Improvement of ECM characteristics by applying ultrasonic vibration. Int. J. Precis. Eng. Manuf..

[17] Ai H., Yan Z., Jiang X., Cheng X., Tian R., Hu Y., Zhang C. (2020). Cathodic Modification, Numerical Simulation and Experimental Investigation on Electrochemical Machining for the Small Inner-walled Ring Groove. Int. J. Electrochem. Sci..

